# Heart failure with preserved ejection fraction: diagnosis, risk assessment, and treatment

**DOI:** 10.1007/s00392-024-02396-4

**Published:** 2024-04-11

**Authors:** Stephan von Haehling, Birgit Assmus, Tarek Bekfani, Elke Dworatzek, Frank Edelmann, Djawid Hashemi, Kristian Hellenkamp, Tibor Kempf, Philipp Raake, Katharina A. Schütt, Rolf Wachter, Paul Christian Schulze, Gerd Hasenfuss, Michael Böhm, Johann Bauersachs

**Affiliations:** 1 Department of Cardiology and Pneumology, University of Göttingen Medical Center, Robert-Koch-Strasse 40, 37075 Göttingen, Germany; 2 German Center for Cardiovascular Research (DZHK), Partner Site Göttingen, Göttingen, Germany; 3 Department of Cardiology and Angiology, Universitätsklinikum Gießen und Marburg, Giessen, Germany; 4 Department of Cardiology and Angiology, Universitätsklinikum Magdeburg, Magdeburg, Germany; 5 Institute of Gender in Medicine, Charité – Universitätsmedizin Berlin, Corporate Member of Freie Universität Berlin, Humboldt-Universität zu Berlin, and Berlin Institute of Health, Berlin, Germany; 6 Charité – Universitätsmedizin Berlin, Corporate Member of Freie Universität Berlin and Humboldt Universität zu Berlin, Berlin, Germany; 7 Department of Cardiology, Angiology and Intensive Care Medicine, Deutsches Herzzentrum der Charité – Medical Heart Center of Charité and German Heart Institute Berlin, Campus Virchow-Klinikum, Berlin, Germany; 8 DZHK (German Centre for Cardiovascular Research), Partner Site Berlin, Berlin, Germany; 9 Berlin Institute of Health at Charité – Universitätsmedizin Berlin, BIH Biomedical Innovation Academy, BIH Charité Digital Clinician Scientist Program, Berlin, Germany; 10 Department of Cardiology and Angiology, Hannover Medical School, Hannover, Germany; 11 I. Medical Department, Cardiology, Pneumology, Endocrinology and Intensive Care Medicine, University Hospital Augsburg, University of Augsburg, Augsburg, Germany; 12 Department of Internal Medicine I, University Hospital RWTH Aachen, Aachen, Germany; 13 Klinik und Poliklinik für Kardiologie, Universitätsklinikum Leipzig, Leipzig, Germany; 14 Department of Internal Medicine I, Division of Cardiology, University Hospital Jena, FSU, Jena, Germany; 15 Kardiologie, Angiologie und Internistische Intensivmedizin, Klinik für Innere Medizin III, Universitätsklinikum des Saarlandes, Saarland University, Homburg, Germany

**Keywords:** Heart failure, Preserved ejection fraction, Diastolic, Diagnosis, Treatment

## Abstract

The aetiology of heart failure with preserved ejection fraction (HFpEF) is heterogenous and overlaps with that of several comorbidities like atrial fibrillation, diabetes mellitus, chronic kidney disease, valvular heart disease, iron deficiency, or sarcopenia. The diagnosis of HFpEF involves evaluating cardiac dysfunction through imaging techniques and assessing increased left ventricular filling pressure, which can be measured directly or estimated through various proxies including natriuretic peptides. To better narrow down the differential diagnosis of HFpEF, European and American heart failure guidelines advocate the use of different algorithms including comorbidities that require diagnosis and rigorous treatment during the evaluation process. Therapeutic recommendations differ between guidelines. Whilst sodium glucose transporter 2 inhibitors have a solid evidence base, the recommendations differ with regard to the use of inhibitors of the renin–angiotensin–aldosterone axis. Unless indicated for specific comorbidities, the use of beta-blockers should be discouraged in HFpEF. The aim of this article is to provide an overview of the current state of the art in HFpEF diagnosis, clinical evaluation, and treatment.

**Graphical Abstract:**

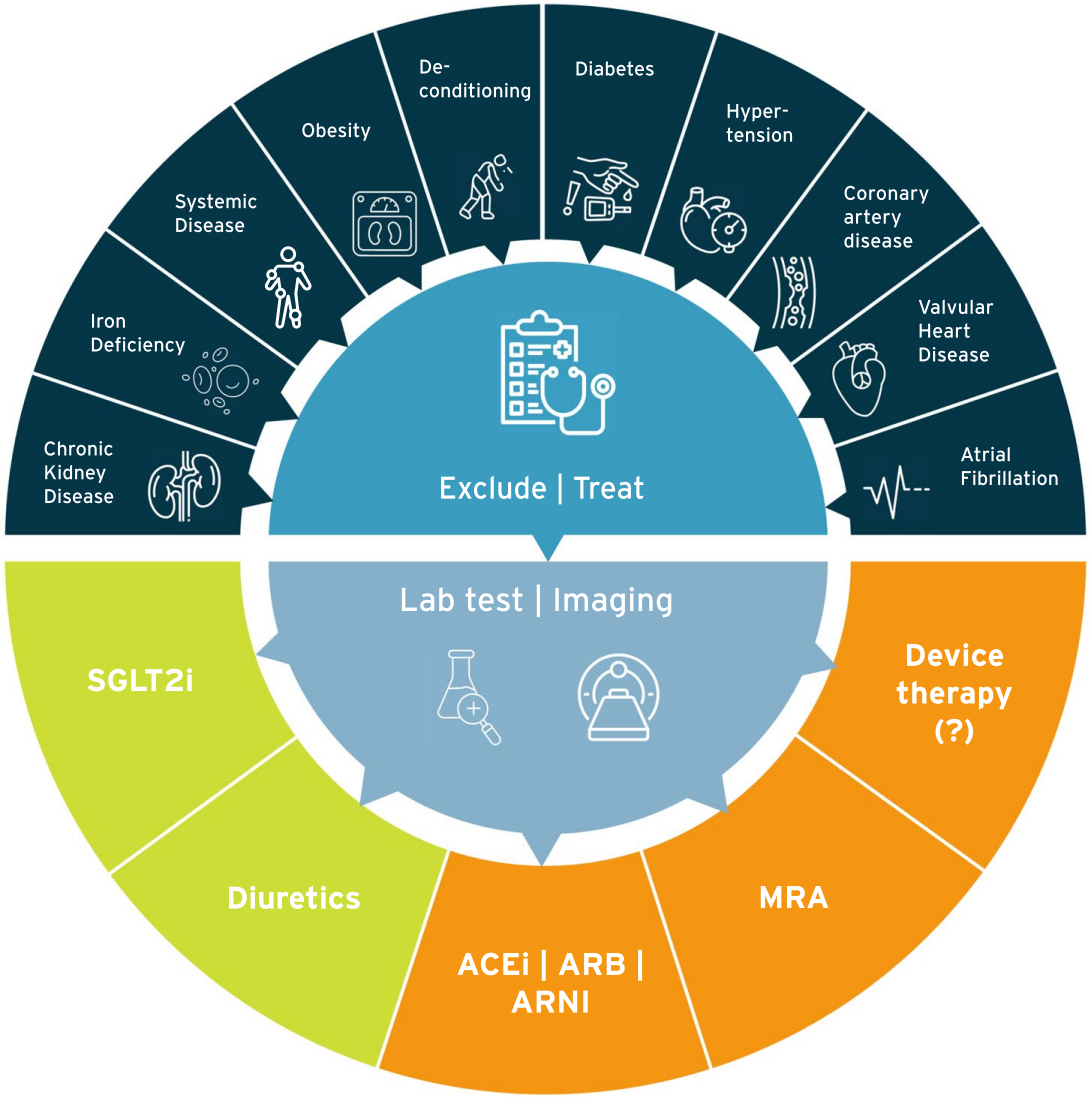

## Introduction

Heart failure (HF) has been called a clinical syndrome, not a disease. The word syndrome stems from the Greek συνδρομή (*syndromḗ*, going together), meaning the joint occurrence of different clinical signs and symptoms. Over the last decades, clinicians have endeavoured to disentangle the HF syndrome yielding the differentiation of HF with reduced (HFrEF), HF with mildly reduced (HFmrEF), and HF with preserved ejection fraction (HFpEF). Even though clinical signs and symptoms overlap grossly between these HF types, the latter type, HFpEF, continues to raise pertinent questions with regard to diagnosis, clinical assessment, and treatment.

It has taken decades to agree on the term HFpEF in international guidelines. In 2008, the European Society of Cardiology (ESC) finally chose to use this term instead of “diastolic HF”, whose use has largely been abandoned over the following years. HFpEF is different from the prototypical form HFrEF for several reasons. It has been suggested that in HFpEF the problem starts in the periphery with hypertension, the metabolic syndrome or the like that spread to the heart, whereas in HFrEF the problem starts in the heart and spreads to the periphery [1]. However, though attractive, this concept has not been proven so far. Women have an overall higher likelihood to develop HFpEF than men (in HFrEF the situation is vice versa), and sex disparities exist (Table 1). From a pathophysiological standpoint, HFpEF develops when the left ventricle (LV) is unable to accept an adequate volume of blood during diastole, at normal diastolic pressures, and at volumes sufficient to maintain an appropriate stroke volume [2]. These abnormalities are caused by a decrease in ventricular relaxation and/or an increase in ventricular stiffness. Till today, there are no established animal models that accurately recapitulate the ventricular complexities leading to HFpEF, yielding difficulties in the development of adequate therapies [3].

**Table 1:** Sex disparities in HFpEF

	**Women**	**Men**
Risk factors	• Older, more likely to be obese (metabolic syndrome) and to have hypertension, and CKD • Anaemia is strongly predictive of all-cause mortality and CV death in HFpEF women • Preeclampsia, autoimmune disease, breast cancer and its treatment	• More likely to have coronary artery disease (more frequently associated with HFrEF), AF, chronic obstructive pulmonary disease (COPD)
Anatomical and pathophysiological differences	• Smaller ventricular chambers and smaller vasculature • Higher risk developing HFpEF as cardiac ageing predisposes women more than men to develop LV concentric remodelling and stiffening • Greater degree of LV dysfunction at rest and exercise as evidence by a lower *e*′, higher *E*/*e*′ ratio, and higher Ed during exercise • Lower diastolic compliance and poorer diastolic reserve • Increased myocardial blood flow and higher myocardial oxygen consumption with prominent lipid metabolism within the myocardium • Greater arterial stiffness and pulse pressure leading to greater pulsatile afterload	• More likely to have eccentric LV remodelling • Lower resistance to ischaemia/reperfusion injury
Biomarkers	• Baseline levels of NT-proBNP and BNP may be higher in women than in men	• NT-proBNP levels may be a more valuable marker of long-term mortality and HF readmission in men compared with women

The aim of this article is to provide clinicians with practical guidance for the diagnosis, clinical evaluation, and treatment of patients with HFpEF, defined as symptomatic HF with a left ventricular ejection fraction (LVEF) ≥ 50%.

## Diagnosis of HFpEF

### Clinical diagnosis

**Fig. 1:**
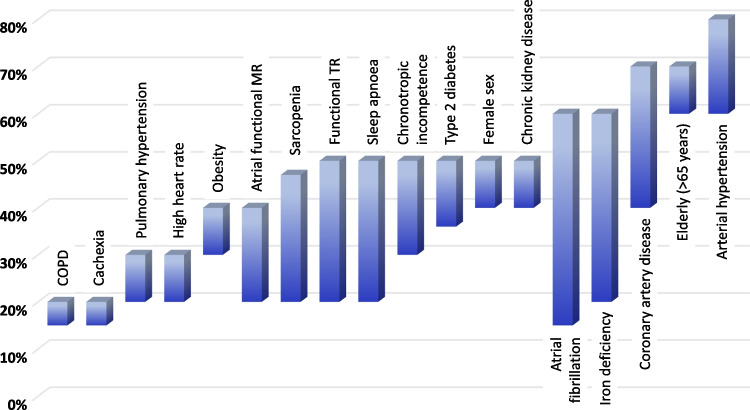
HFpEF phenotypes with upper and lower boundary of the estimated prevalence values. Modified from Anker et al. [4] COPD, chronic obstructive pulmonary disease; MR, mitral regurgitation; TR, tricuspid regurgitation

**Fig. 2:**
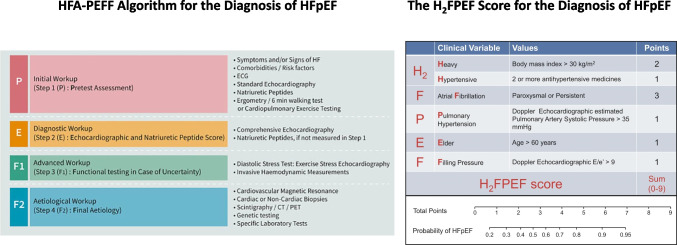
Clinical scores estimating the likelihood of a HFpEF diagnosis. Modified from Pieske et al. [5] (left panel) and Reddy et al*. *[7] (right panel). CT, computed tomography; ECG, electrocardiogram; PET, positron emission tomography

**Fig. 3:**
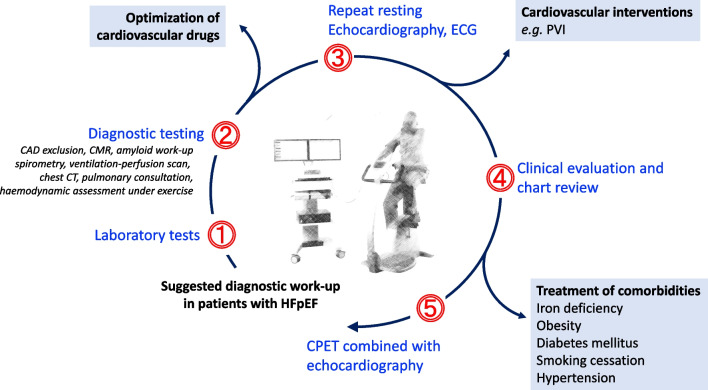
Components of assessing the contributors to dyspnoea in patients with confirmed diagnosis of HFpEF according to the HFA-PEFF or the H_2_FPEF score. Cut-off values were ≥ 5 and ≥ 6, respectively. Modified from Verwerft et al. [8] CAD, coronary artery disease; CMR, cardiac magnetic resonance imaging; CPET, cardiopulmonary exercise test; CT, computed tomography; ECG, electrocardiogram; PVI, pulmonary vein isolation

The clinical diagnosis of HFpEF remains challenging due to its heterogeneous aetiology (see “Evaluating the presence of systemic disease”) that recently sparked the idea of different HFpEF phenotypes that may require different treatment strategies with much focus on patients’ comorbidities (Fig. 1) [4]. The introduction of two different diagnostic tools has greatly enhanced our ability to diagnose HFpEF in recent years. The first, the HFA-PEFF algorithm offers a structured approach to the diagnosis of HFpEF by assessing predisposing risk factors, clinical signs and symptoms, objective evidence of cardiac dysfunction, and functional and structural abnormalities [5]. Each criterion is assigned a score, reflecting the probability of HFpEF and its impact on prognosis [6]. A second, recently proposed diagnostic tool is the H_2_FPEF score [7]. Both algorithms are summarized in Fig. 2. Finally, a diagnostic algorithm has recently been proposed for patients with HFpEF to identify comorbidities involved in the development of dyspnoea and/or other symptoms suggestive of HFpEF as shown in Fig. 3 [8].

Factors enhancing the pre-test probability for the diagnosis of HFpEF encompass female sex, advanced age, hypertension, obesity, chronic kidney disease (CKD), diabetes mellitus (DM), and atrial fibrillation (AF). These factors indicate the development of diastolic dysfunction and LV hypertrophy [9]. When patients with typical symptoms such as exertional dyspnoea, fatigue, and reduced exercise tolerance present alongside these risk indicators, HFpEF should be suspected. Clinical signs and symptoms of HFpEF resemble those of other forms of HF, including peripheral oedema, elevated jugular venous pressure, and pulmonary congestion on auscultation or chest radiography [10]. Whilst these findings are not specific to HFpEF, they provide supportive evidence for the diagnosis. Furthermore, orthopnoea and paroxysmal nocturnal dyspnoea are commonly reported symptoms in patients with HFpEF, often resulting from elevated LV filling pressures during recumbency [7].

Objective evidence of cardiac dysfunction is required to confirm the diagnosis of HFpEF. Elevated levels of natriuretic peptides, such as B-type natriuretic peptide (BNP) and N-terminal pro-B-type natriuretic peptide (NT-proBNP), are suggestive of HFpEF, particularly in patients with predisposing risk factors and clinical signs of HF [11]. However, it is essential to consider other causes of elevated natriuretic peptides, such as acute coronary syndromes, valvular heart disease, AF, and CKD (see “Chronic kidney disease and hyperkalaemia”). Echocardiography remains the cornerstone of functional and structural assessment in patients with suspected HFpEF [12]: LVEF is typically preserved (≥ 50%), left atrial volume index (LAVI) is increased, and there is evidence of diastolic dysfunction leading to elevated cardiac filling pressures [12]. The presence of a hypercontractile phenotype has been suggested to exist in opposition to a normocontractile phenotype, with the former characterized by an LVEF > 60–70% and the latter by an LVEF below this range [13]. Diastolic dysfunction is defined as a shift in the LV end-diastolic pressure–volume (LVEDPVR) relation, and echocardiographic measurements are merely non-invasive approaches. Thus, invasive haemodynamic assessments, such as measuring left ventricular end-diastolic pressure (LVEDP) under exercise or hand-grip testing and right heart catheterization with or without exercise, can offer a more detailed understanding of diastolic function and provide valuable insights into the diagnosis of HFpEF [7]. Moreover, it is important to acknowledge the value of functional testing over plain coronary angiography, because non-obstructive coronary artery disease (CAD) is over-represented in HFpEF, placing more emphasis on coronary microvascular dysfunction [9, 14]. Cut-off values reflecting elevated LV filling pressures are summarized in Table 2, which also shows differences between ESC guidelines for the diagnosis and treatment of HF [10] and the American guidelines issued jointly by the American College of Cardiology/American Heart Association/Heart Failure Society of America (ACC/AHA/HFSA) [15]. Whether the one or the other algorithm is superior in clinical practice is still a matter of clinical studies [16, 17].

**Table 2:** Comparison of major differences in the ESC and American HF Guidelines regarding HFpEF diagnosis and treatment

		ESC guideline 2021 with clinical update 2023	ACC/AHA/HFSA guideline 2022
	Diagnosis		
Simplified diagnostic approach		Symptoms ± signs^a^	
		LVEF ≥ 50%	LVEF ≥ 50%
		Objective evidence of cardiac structural and/or functional abnormalities consistent with the presence of LV diastolic dysfunction/raised LV filling pressures, including raised natriuretic peptides^b^	Evidence of spontaneous (at rest) or provokable (*e.g.* during exercise, fluid challenge) increased LV filling pressures (*e.g.* elevated natriuretic peptide, non-invasive and invasive haemodynamic measurement)
	Useful diagnostic criteria of raised LV filling pressure		
Echocardiographic criteria	Structural criteria	LAVI > 34 ml/m^2^ (sinus rhythm)^e^ or > 40 ml/m^2^ (AF)	Increase in LA size and volume (LAVI) ≥ 29 ml/m^2^
		LV mass index ≥ 95 g/m^2^ (female), ≥ 115 g/m^2^ (male) Relative wall thickness (RWT) > 0.42	Increase in LV mass (LV mass index) > 116/95 g/m^2^ Relative wall thickness (RWT) > 0.42 LV wall thickness ≥ 12 mm
	Functional criteria	*E*/*e*′ ratio > 9 (rest) or ≥ 15 (peak exercise) GLS < 16% Additional criteria:^f^ • Mitral *E* velocity < 90 cm/s • Septal *e*′ velocity < 9 cm/s	*E*/*e*′ ≥ 15 GLS < 16% Additional criteria: • Septal *e*′ velocity < 7 cm/s • Lateral *e*′ velocity < 10 cm/s
	Other criteria	PA systolic pressure > 35 mmHg; tricuspid regurgitation velocity > 2.8 m/s (rest) or > 3.4 m/s (peak exercise)	• TR velocity > 2.8 m/s • Estimated PA systolic pressure > 35 mm Hg
Invasive testing		PCWP ≥ 15 mmHg (at rest) or ≥ 25 mmHg (with exercise) LV end-diastolic pressure ≥ 16 mmHg (at rest)^c^	Haemodynamics at rest or with exercise, with assessment of filling pressures (PCWP or LV end-diastolic pressures, pulmonary artery (PA) pressures, stroke volumes, and cardiac output)
Natriuretic peptides		NT-proBNP > 125 (sinus rhythm) or > 365 (AF) pg/ml BNP > 35 (sinus rhythm) or > 105 (AF) pg/ml	NT-proBNP > 125 pg/ml; BNP ≥ 35 pg/ml
Scores		HFA-PEFF, H_2_FPEF	H_2_FPEF (HFA-PEFF)
	Treatment recommendations		
Hypertension management (management of comorbidities in general in the ESC HF guideline)		I (C)	I (C)
Treatment of AF to improve symptoms			IIa (C)
Diuretics in congested patients		I (C)	I (C)
SGLT2i		I (A)	IIa (B)
MRAs			IIb (B)^d^
ARBs			IIb (B)^d^
ARNi			IIb (B)^d^

^a^Signs may not be present in the early stages of HF (especially in HFpEF) and in optimally treated patients

^b^For the diagnosis of HFpEF, the greater the number of abnormalities present, the higher the likelihood of HFpEF

^c^Right heart catheterization may be considered in selected patients with HFpEF to confirm the diagnosis

^d^Particularly among patients with LVEF on the lower end of this spectrum

^e^This cut-off is taken from Table 9 “Objective evidence of cardiac structural, functional and serological abnormalities consistent with the presence of left ventricular diastolic dysfunction/raised left ventricular filling pressures” of the ESC guidelines. In contrast, in the running text of the corresponding guideline section, a cut-off value of > 32 ml/m^2^ is given for the LA volume index

^f^Additional criteria are provided in the running text of the ESC guideline

^g^Additional criteria are provided in Appendix 3 “Appendix for Table 3 and 4: Suggested Thresholds for Structural Heart Disease and Evidence of Increased Filling Pressures” of the 2022 ACC/AHA/HFSA guideline

Exercise testing, including cardiopulmonary exercise testing (CPET), allows for the assessment of functional capacity, ventilatory efficiency, and gas exchange abnormalities during exercise, which may help to identify exercise-induced elevations in pulmonary artery and LV filling pressures, particularly in patients with unexplained dyspnoea [18]. CPET can also be used to differentiate HFpEF from other causes of exercise intolerance, such as pulmonary diseases or deconditioning [19]. Finally, right heart catheterization at exercise, for a meaningful number of patients, allows for the direct measurement of LV filling pressures, pulmonary artery pressures, and cardiac output, providing valuable haemodynamic information to support the diagnosis of HFpEF [20].

### Biomarkers

Circulating concentrations of the natriuretic peptides BNP and NT-proBNP are elevated in HFpEF populations, albeit to a lesser extent than in patients with HFrEF [21, 22]. A substantial number of patients with HFpEF present with mildly elevated natriuretic peptide levels located within a diagnostic grey zone and possible differences between men and women (Table 1). Comorbidities have significant influence on natriuretic peptide levels, particularly AF, CKD, and obesity [22]. Whilst AF and CKD are associated with increased natriuretic peptide concentrations, obesity associates with lower levels. In patients with a body mass index (BMI) ≥ 30 kg/m^2^, it has been suggested to reduce diagnostic natriuretic peptide cut-off levels by 50% [22]. ESC and American HF guidelines and recent consensus documents suggest the use of natriuretic peptides for diagnosing elevated filling pressures in HFpEF in the acute and non-acute settings as shown in Table 2 [10, 15, 22]. In the work-up of patients with suspected acute HF, the same cut-off levels as shown in Table 2 should be used [22]. Elevated BNP and NT-proBNP concentrations are integrated as major or minor criteria in the HFA-PEFF score (Fig. 2) using different cut-off levels for patients in sinus rhythm and AF.^5^ Of note, elevated natriuretic peptide concentrations have been used as inclusion criteria in the PARAGON, DELIVER, and EMPEROR-Preserved trials (see “SGLT2 inhibitors”) [23–25].

The performance of both biomarkers has been questioned in the diagnosis of HFpEF in outpatients with unexplained dyspnoea. Indeed, up to one-third of patients with HFpEF and an elevated pulmonary capillary wedge pressure (PCWP) at rest were found to display a BNP value below the diagnostic threshold [26]. In patients with exercise-induced dyspnoea assessed by CPET, one-third was diagnosed with HFpEF despite circulating NT-proBNP concentrations < 125 ng/l [27]. These patients had higher risk of HF events than patients without HFpEF and with normal NT-proBNP. From a pathophysiological perspective, a small ventricle size in a hypertrophic ventricle, a classical feature of HFpEF, counteracts increased wall stress, which is the main trigger for natriuretic peptide release. The imperfection of natriuretic peptides in the diagnosis of HFpEF is also reflected by not qualifying as a variable for the H_2_FPEF score [7]. In fact, the HFA-PEFF score may be consistent with a diagnosis of HFpEF, even when natriuretic peptide levels are below the given thresholds (Fig. 2) [5].

### Imaging in HFpEF

Among the various imaging techniques, transthoracic echocardiography (TTE) is most valuable in providing information about cardiac structure and function such as LV hypertrophy, LV mass, and diastolic function [5, 10]. Tissue Doppler imaging (TDI) offers insight into diastolic dysfunction by measuring early diastolic relaxation velocity of the mitral annulus (*e*′). Speckle-tracking echocardiography (STE) enables assessment of myocardial strain and left atrial (LA) function [28]. These measurements aid estimating elevated LV end-diastolic pressure (LVEDP), using parameters such as the *E*/*e*′ ratio derived from TDI [28]. Whilst exercise echocardiography can help in certain cases by revealing exercise-induced changes in diastolic function that might not be apparent at rest, its widespread diagnostic use remains controversial with some studies highlighting its limitations in routine use and the limited additional value at increasing the sensitivity for diastolic dysfunction [5, 17].

Cardiac magnetic resonance (CMR) enables high-resolution imaging and detailed tissue characterization [30]. It allows accurate functional assessment, myocardial strain, and fibrosis imaging using techniques such as late gadolinium enhancement (LGE) and T1/T2 mapping. Advanced techniques like 4D flow permit visualization of intracardiac blood flow patterns, whilst parametric mapping methods like native T1 and extracellular volume (ECV) mapping reveal myocardial tissue properties [29, 30]. Tissue characterization by CMR can aid in excluding storage diseases like amyloidosis (see “Evaluating the presence of systemic disease”) [31] and can also help to exclude other potential causes of HF, such as infiltrative cardiomyopathies or myocarditis. Like TTE, CMR imaging is now possible during exercise in order to assess dynamic changes in ventricular function and perfusion during exercise [32]. It can also help in the assessment of elevated LVEDP through the evaluation of LA volumes and function, which are related to diastolic function and filling pressures [33]. Computed tomography (CT) can be useful for assessing cardiovascular (CV) anatomy and detecting coronary artery calcification and potential pulmonary embolism. However, its role in HFpEF is limited due to its low sensitivity for detecting subtle changes in cardiac function [10]. Finally, positron emission tomography (PET) may provide insight into myocardial perfusion, metabolism, and inflammatory burden. It may have a potential role in assessing HFpEF, especially in understanding its pathophysiology, but its clinical use remains limited due to cost and availability [34].

## Clinical evaluation and risk assessment

### Evaluating the presence of systemic disease

The majority of HFpEF cases are attributable to common CV risk factors and comorbidities like hypertension, DM, and obesity. Still, the possibility of non-CV systemic aetiology should be considered in situations outlined in Table 3, in which secondary HFpEF may be present [5]. Indeed, most systemic diseases have extracardiac symptoms, such as polyneuropathy in ATTR amyloidosis, macroglossia in AL amyloidosis, and stroke-like episodes or seizures in mitochondrial disease. Specific systemic diseases that may be present in HFpEF are summarized in Table 4. Comorbidities and secondary HFpEF as a result of systemic disease should be assessed using a structured approach as outlined in Fig. 3.

**Table 3:** Criteria for considering a non-cardiovascular cause of HFpEF

Young age
Family history of cardiomyopathies/heart failure
No or only minor classical risk factors/comorbidities
Inadequate left ventricular hypertrophy (> 13 mm)
Specific echocardiographic strain patterns (e.g. amyloidosis)
Significant ventricular arrhythmias
Severe right heart dilatation
Significant scar tissue (in cardiac MRI)
Regional wall motion abnormalities

**Table 4:** Potential systemic aetiologies for HFpEF syndrome

Autoimmune diseases	Systemic lupus erythematosus, scleroderma, dermato/polymyositis, hypereosinophilic syndrome
Infiltrative diseases	Direct infiltration and metastases, carcinoid, Paget’s disease
Metabolic and storage diseases	Amyloidosis, sarcoidosis, haemochromatosis, Fabry disease (and other lysosomal storage diseases)
	Glycogen storage disease (Pompe, Danon)
	Sphingolipidosis (Gaucher)
	Mucopolysaccharidosis (Hunter, Hurler, Scheie)
Neuromuscular disorders	Friedreich ataxia
Mitochondrial diseases	MELAS, MERFF (and others)
Malformation syndromes	Noonan, Leopard, Costello

**Fig. 4:**
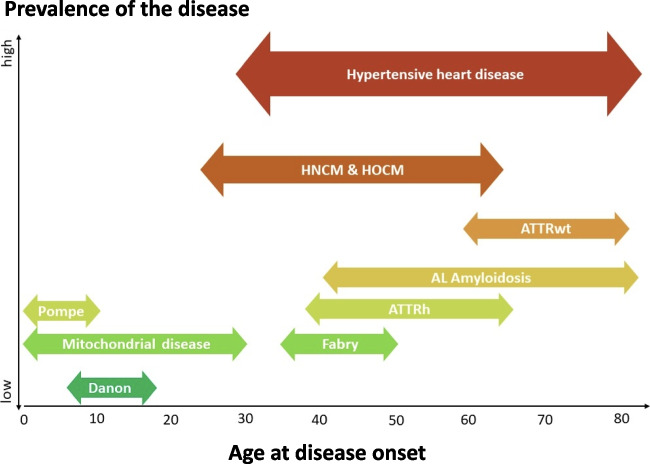
Relative prevalence and HFpEF patients’ age at onset caused by systemic disease. Modified from Kubo et al*. *[154] ATTRwt, wild-type transthyretin amyloidosis; ATTRh, hereditary transthyretin amyloidosis; HNCM, hypertrophic non-obstructive cardiomyopathy; HOCM, hypertrophic obstructive cardiomyopathy

Cardiac amyloidosis is among the comparatively frequent systemic diseases in secondary HFpEF. It results from misfolded proteins that accumulate in various organs: light-chain proteins in AL amyloidosis [35] and transthyretin either in wild-type (ATTRwt) or hereditary (ATTRh) amyloidosis. Diagnostic extracardiac “red flags” in ATTR amyloidosis are bilateral carpal tunnel syndrome, ruptured biceps and tendons, lumbar spinal stenosis, and polyneuropathy, whereas macroglossia, nephrotic syndrome, and periorbital haematoma frequently occur with AL amyloidosis [36]. Cardiac involvement diagnosis is based on LV hypertrophy and typical strain patterns on TTE (see “Imaging in HFpEF”). If suspected, immediate blood testing for light-chain abnormalities should be performed, in addition to imaging with CMR or technetium-diphosphonate (DPD) scintigraphy, in cases of ATTR amyloidosis followed by genetic testing. Treatment of ATTR cardiomyopathy with tafamidis reduces disease progression and HF hospitalization in New York Heart Association classes I and II, whereas AL amyloidosis requires haematological treatment [10]. Fabry disease is a rare X-linked lysosomal storage disorder that manifests as hypertrophic cardiomyopathy, cryptogenic stroke, or end-stage renal disease. Extracardiac symptoms include angiokeratoma, vertigo, and gastrointestinal issues. Enzyme replacement therapy has demonstrated benefits. Finally, mitochondrial disorders [37], which are rare genetic diseases affecting organs reliant on mitochondrial oxidative metabolism, can lead to cardiomyopathy and/or conduction defects. The age of disease onset can aid in diagnosis, with many genetic disorders presenting early in life (Fig. 4).

### Diabetes mellitus

Patients with DM exhibit a 2–fourfold increase in the risk of developing HF [38–41], whose aetiology embraces coronary artery disease, arterial hypertension, direct or indirect effects of hyperglycaemia, and obesity-associated factors [42, 43]. Likewise, longer duration of DM, increased BMI, and CKD are associated with HF [44, 45]. A large European registry found that ~ 36% of all outpatients with HF have DM [46], with numbers rising to 50% in decompensated patients [47]. HFrEF and HFpEF are equally common in individuals with DM, but HFpEF is more likely to remain undetected [48–50]. In the Atherosclerosis Risk In the Community (ARIC) study, worsening dysglycaemia was associated with increased LV mass, worse diastolic function, and subtle reduction in LV systolic function. For every 1% increase in glycated haemoglobin, LV mass was higher by 3.0 g, *E*/*e*′ by 0.5, and global longitudinal strain by 0.3% suggesting dysglycaemia to contribute to subclinical impairment in cardiac structure and function [51]. It is important to acknowledge that the coexistence of DM and HF is associated with increased CV mortality [52, 53]. In patients with acute decompensated HF, the presence of DM is associated with increased intrahospital [47] and 1-year mortality [54] as well as rehospitalization rate [47]. These results were confirmed also in the EMPEROR-Preserved and DELIVER trials [24, 25]. Altogether, individuals with DM should be regularly assessed for the presence of typical HF signs and symptoms. If HF is suspected, additional diagnostic tests are recommended (Table 4). Vice versa, all patients with HF should be screened for the presence of DM.

### Atrial fibrillation

According to the Framingham study, 55% of patients with HFrEF have AF during their lifetime; the corresponding number for HFpEF reaches 62%. Of these, 32% have AF before the diagnosis of HFpEF, in 18% the two diagnoses coincide, and 12% receive the diagnosis of AF after the diagnosis of HFpEF [55]. AF is independently associated with an increased risk of HF hospitalization and death in HFpEF and HFrEF, but the association in HFpEF is stronger [56]. The presence of AF is the most relevant indicator that HF symptoms (*e.g.* dyspnoea) are caused by HFpEF and therefore a prominent component in the H_2_FPEF algorithm (Fig. 2) [7]. The HFA-PEFF algorithm uses natriuretic peptide testing instead of AF [5]. The response to HF treatment with the SGLT2i empagliflozin [23] and dapagliflozin [57] is similar in patients with and without AF. In patients with clinically stable HF in the CABANA trial that predominantly recruited patients with HFpEF, treatment of AF pulmonary vein isolation (in comparison to drug therapy) was associated with clinically relevant improvements in survival, freedom from AF recurrence, and quality of life [58].

### Chronic kidney disease and hyperkalaemia

CKD affects at least 40–50% of all patients with HF with somewhat higher prevalence in HFpEF than in HFrEF [59]. In the DELIVER trial, 49% of patients randomized to dapagliflozin or placebo had an estimated glomerular filtration rate (eGFR) < 60 ml/min/1.73 m^2 ^[60]. In the EMPEROR-preserved trial, 53.5% of all patients had CKD, defined as eGFR < 60 ml/min/1.73 m^2^ or urine albumin to creatinine ratio > 300 mg/g [61]. The effect of CKD on mortality appears to be less pronounced in HFpEF than in HFrEF [59]. CKD itself has been associated with a higher incidence of HFpEF, as renal dysfunction can contribute to fluid overload and increased cardiac stress [62]. Hyperkalaemia has been associated with worse prognosis and an increased likelihood of suboptimal medical therapy, particularly with regard to inhibitors of the renin–angiotensin–aldosterone axis such as mineralocorticoid receptor antagonists (MRAs). Other predisposing risk factors of hyperkalaemia include DM, advanced age, CKD, and high baseline potassium values. The potassium binder patiromer has shown efficacy in patients with HF both in lowering potassium levels and in enabling MRA therapy [63], and subanalyses of large trials suggest similar effectiveness for sodium zirconium cyclosilicate [64], but no trial data are available specifically for patients with HFpEF and thus further investigations are needed.

### Iron deficiency

The ESC guidelines for the management of HF define iron deficiency (ID) irrespective of the respective type as present when serum ferritin is < 100 ng/ml or when serum ferritin is of 100–299 ng/ml with transferrin saturation (TSAT) < 20%.^10^ Further, the guidelines recommend that “all patients with HF are periodically screened for anaemia and ID”. Both points are important because treatment studies showing the effectiveness of intravenous iron repletion with ferric carboxymaltose and ferric derisomaltose are available for patients with HFrEF and HFmrEF only. Using these criteria, several studies investigated ID in patients with HFpEF. A large meta-analysis published in 2019 including data of 1424 patients with HFpEF found a prevalence of ID of 59% [65]. Later studies either confirmed these data at a prevalence of 57.5% or found higher values at 75%. Overall, the prevalence of ID appears to be higher in patients with decompensated as compared to compensated HF [66, 67]. Since iron is crucially involved in erythropoiesis and present in all enzymes of the respiratory chain in myocardium and skeletal muscle, there is a high likelihood of ID having an impact on exercise capacity. Study results in patients with HFpEF and ID, however, have been inconsistent: ID was associated with lower peak oxygen consumption in three of four studies, with lower 6-min walking test distance in two of three studies and with lower quality of life in two studies [65]. Barandiarán Aizpurua et al. [68] found a significant association between ID and reduced exercise capacity, but this was lost after multivariable adjustment. Using data from 300 patients with HFpEF, the latter study found that patients with ID have worse prognosis with a higher likelihood of reaching the combined endpoint of all-cause mortality or hospitalization for HF after 4 years of follow-up. The results of the double-blind, randomized FAIR-HFpEF trial are currently awaited [69]. Retrospective analyses suggest functional improvement after application of ferric carboxymaltose [70].

### Valvular heart disease

Valvular heart disease is common in HFpEF. Functional tricuspid regurgitation (TR) affects 20–50%, [4, 71, 72] functional mitral regurgitation (MR) up to 20% of all patients with HFpEF [48, 73]. Isolated functional TR refers to atrial origin and is often associated with AF [74]. Patients with severe TR in HF experience symptoms such as dyspnoea and venous congestion [74], and TR has been identified as an independent predictor of adverse outcomes and mortality [75, 76]. Transcatheter tricuspid valve intervention (TTVI) has gained attention recently as a safer alternative to isolated tricuspid valve surgery [10, 77, 78]. Studies have shown the safety and positive impact of TTVI on quality of life, hospitalization, and survival, especially in HFpEF [78–81]. Ongoing trials aim to validate this new therapy. Similarly, functional MR is associated with increased cardiovascular morbidity and mortality and is often caused by atrial distention [73]. Early studies and registry analyses suggest that transcatheter mitral valve interventions can be safe and provide functional, clinical, and symptomatic benefits, but this needs confirmation through randomized controlled trials [82].

### Wasting and frailty

Both cachexia and sarcopenia are prevalent conditions in patients with HF and can increase the risk of developing frailty [83]. Cachexia refers to the involuntary weight loss that occurs in the presence of chronic illnesses like HF, whilst sarcopenia specifically refers to the loss of skeletal muscle mass and impaired skeletal muscle function [84]. These terms, occasionally accompanied by the loss of bone mineral density [85], collectively fall under the category of body wasting. Sarcopenia may, but does not necessarily, precede cachexia [86]. A recent meta-analysis has shown that sarcopenia is more prevalent in patients with HFrEF than HFpEF (28 vs. 18%) and has an overall higher prevalence among patients HF in Asia (35%) than in Europe (31%) or the Americas (25%) [87]. In patients with HFpEF, sarcopenia is an independent predictor of death [88]; however, this association may be less pronounced in HFpEF than in HFrEF [89]. Clinical implications of sarcopenia in HFpEF embrace lower 6-min walking distance, lower peak oxygen consumption on spiroergometry, and lower quality of life [90]. Cytokine expression patterns contributing to sarcopenia development appear to differ between HFrEF and HFpEF [91]. Finally, according to a recent ESC position paper [92], frailty is defined as “a multidimensional dynamic state, independent of age, that makes the individual with HF more vulnerable to the effect of stressors”. It affects up to 45% of all patients with HF with body wasting being one of the chief contributing factors to become frail [10]. Multidisciplinary interventions including exercise training and nutritional support might be beneficial for affected patients [93, 94].

## Treatment

The treatment of HFpEF requires a holistic approach that identifies potentially treatable comorbidities as discussed above and in Fig. 3. Apart from pharmacological treatment discussed below and in Table 3, patient counseling encompasses a holistic approach addressing diet, physical activity, and psychological well-being [95, 96]. Dietary recommendations often focus on sodium restriction to manage fluid retention and optimize blood pressure control. Engaging in regular, tailored physical activity is encouraged, emphasizing activities suitable for the individual’s fitness level. A multidisciplinary approach involving psychology is essential, recognizing the emotional impact of HF. Counseling may address stress management, coping strategies, and fostering a positive mindset to enhance overall quality of life.

### ACEi, ARBs, and ARNI

Interpretation of the results of trials blocking the renin-angiotensin-system (RAS) differs significantly between ESC and American HF guidelines (Table 3). Whilst in HFmrEF, there is general agreement that RAS blockade is indicated based on subanalyses of large randomized trials, recommendations for RAS blockade in HFpEF are currently provided in the American HF guidelines only [15]. None of the trials in HFpEF with the angiotensin-converting enzyme inhibitor (ACEi) perindopril, or the angiotensin-receptor blockers (ARBs) candesartan and irbesartan, met its primary endpoint [97–99]. In case of candesartan, a retrospective analysis of the CHARM program revealed that this ARB reduces outcomes only in HFrEF and HFmrEF, but not HFpEF [100]. Nevertheless, many patients with HFpEF are treated with these drugs for the management of comorbidities such as hypertension and CKD.

In the PARAGON trial in patients with an LVEF > 45%, the angiotensin-receptor neprilysin inhibitor (ARNI) sacubitril/valsartan did not significantly reduce the primary endpoint of CV death and total HF hospitalizations [23]. It is interesting to acknowledge a number of secondary analyses that provided valuable insight into subgroups of patients. One subanalysis of the PARAGON-HF trial suggested that women with HFpEF are more likely to benefit from ARNI treatment than men [101]. In both sexes, there seemed to be effectiveness in HFmrEF, and higher LVEF values were not associated with benefit when compared to valsartan. However, in a pre-specified participant-level pooled analysis of the PARAGLIDE-HF and PARAGON-HF trials in patients with LVEF > 45% and a recent HF decompensation, sacubitril/valsartan reduced the risk of worsening HF and CV death significantly and quite early after randomization [102]. Benefits of sacubitril/valsartan were more pronounced in patients with LVEF ≤ 60% (*p* for interaction = 0.021). Thus, ARNI treatment may be of value in HFpEF patients with LVEF ≤ 60% and a recent decompensation [103]. Similarly, the STRONG-HF trial demonstrated that after a hospitalization for acute HF rapid uptitration of oral medications including ACEi/ARB/ARNI and close follow-up did reduce mortality and HF rehospitalization as well as quality of life independent of LVEF [104]. These results suggest that patients with HFpEF should be treated with RAS blockade at least after a worsening HF event.

### Beta-blockers

Βeta-blockers are widely prescribed in patients with HFpEF, although evidence for beneficial effects in these patients is poor. In a large cohort of 19,083 patients with HFpEF from the Swedish HF registry, 83% were on β-blockers [105]. This may in part be related to comorbidities such as AF, hypertension, or previous myocardial infarction (MI). However, β-blockers are no longer first choice for hypertension, and in this cohort AF and post-MI prevalence was lower than 50% and 30%, respectively. Therefore, many patients may receive β-blockers primarily under the intention to treat HFpEF, driven partially by the pathophysiological concept that LV filling may be improved by longer diastole at lower heart rates. On the contrary, myocardial relaxation is supported by sympathetic tone-mediated cyclic AMP, which is decreased by β-blockers and prolonged diastolic filling results in increased LV end-diastolic volume and wall stress. Moreover, a significant number of patients with HFpEF suffer from chronotropic incompetence, which may worsen further under β-blocker treatment [106].

In the SENIORS trial [107], nebivolol showed a reduction of combined all-cause mortality or CV hospitalization in patients with HFrEF, HFmrEF, and HFpEF with no differences between groups. In registries or secondary analyses from randomized trials, however, results are not consistent. A propensity score–matched cohort study using the Swedish Heart Failure Registry showed a statistically significant, numerically larger survival of 45 compared to 42% after 5 years of β-blocker treatment in HFpEF patients (HR of 0.93, *p* = 0.04) [105]. However, survival free from HF hospitalization was not different between both groups suggesting that HF hospitalization may at best not improve with β-blockers. This would be consistent with a recent secondary analysis of the TOPCAT trial [108]. Here, β-blocker use was associated with a higher risk of HF hospitalization among patients with HFpEF and a LVEF ≥ 50% (HR 1.74, *p* < 0.01). Thus, without further randomized data to treat or not to treat HFpEF patients with β-blockers is an individual decision. There is no recommendation to treat HFpEF patients with β-blockers and it seems appropriate to stop β-blocker treatment to check for symptomatic improvement and increased exercise capacity [107].

### MRAs

Activation of the mineralocorticoid receptor (MR) in CV cells such as cardiomyocytes, endothelial, and vascular smooth muscle cells as well as myeloid cells is a well-described phenomenon contributing to the pathophysiology of HF independent of LVEF [109, 110]. In addition to the data in HFrEF where MR antagonists (MRA) have a class IA indication according to all HF guidelines, numerous pre-clinical and clinical studies have demonstrated beneficial effects of MRAs in HFpEF and HFmrEF [110]. The class IIB recommendation for use of spironolactone in ESC and American guidelines is based on a post hoc analysis of the TOPCAT trial (spironolactone in HF with LVEF ≥ 45%), which suggested that in a subgroup of patients with LVEF 44–49%, spironolactone reduced the risk of the primary endpoint (CV death, HF hospitalization, or resuscitated sudden death), mostly due to a reduction in CV mortality with spironolactone [111].

In patients with HFpEF, European and American HF guidelines diverge [112]: Whilst the latter assigns a IIB recommendation to spironolactone for selected patients with HFpEF, particularly those at the lower end of the LVEF spectrum, to decrease hospitalizations, such statement has not been included in the 2021 ESC guideline. Support for the recommendation for spironolactone in HFpEF comes from subanalyses of the TOPCAT trial: Particularly in American patients, spironolactone reduced the primary endpoint [113]. Moreover, patients included on the basis of elevated natriuretic peptide levels at baseline as a clear indictor for manifest HF did benefit from the use of spironolactone [114]. To further support the use of spironolactone in HFpEF, it needs to be acknowledged that the drug exerts beneficial effects on several comorbidities such as hypertension, LV hypertrophy, CV fibrosis, fluid retention, right HF, (re)occurrence of AF, and the metabolic syndrome [110]. Three randomized controlled trials with MRAs are currently ongoing in patients with HFpEF and HFmrEF, including SPIRIT-HF (EudraCT 2017–000697-11) and SPIRRIT (NCT02901184) using spironolactone as well as FINEARTS investigating the novel non-steroidal MRA finerenone against placebo.

### SGLT2 inhibitors

SGLT2i have emerged as a novel treatment option for patients with HF, irrespective of underlying subtype [24, 25, 115–122]. In patients with HFpEF, both dapagliflozin and empagliflozin have shown beneficial effects on CV mortality and HF hospitalization in EMPEROR-Preserved and DELIVER, [24, 25] yielding a class I A recommendation in the 2023 focused update of the 2021 ESC HF guidelines [123]. Further prognostic benefits on the progression of CKD were shown in the EMPA-KIDNEY and DAPA-CKD trials [124, 125]. Of note, in all studies, the impact on CV hospitalization rates outperformed mortality–lowering benefits of SGLT2i.

SGLT2i act through inhibition of the SGLT2 in the proximal renal tubulus leading to increased glucosuria and diuresis [120, 121, 125], effects described in patients with chronic stable and acute decompensated HF [24, 25, 115–121, 126–128]. SGLT2i do not increase sympathetic or neurohumoral drive in patients with HF but do enhance effects of loop diuretics [121]. A definite cardiac mechanism of action remains elusive; peripheral effects have been linked to increased glucosuria, improved diuretic responsiveness, and renal protection [121, 129, 130]. Indeed, SGLT2i induce glucosuria associated with a caloric deficit of 250–300 kilocalories per day [131]. This process increases cardiac free fatty acid oxidation and circulating ketone body levels including beta-hydroxybutyrate, an alternative energy source that can be utilized by cardiomyocytes [132]. Whilst other hypotheses have been proposed, enhanced ketone body metabolism under SGLT2i may in part explain the beneficial effects of SGLTi in HF through a shift in cardiac energy substrates utilization [131]. Of note, whilst initial evidence from animal studies and patient cohorts supported this hypothesis, a recent randomized trial in subjects with HF showed that 12 weeks of treatment with empagliflozin 10 mg did not affect cardiac energy metabolism assessed by NMR and did not result in changes in circulating metabolites compared to placebo [133]. Thus, the beneficial cardiac effects of SGLT2i might be mediated through secondary effects without metabolic mediators.

### Device therapy

Elevated LA pressure during rest or exercise is considered a key pathophysiological feature of HFpEF. Elevated LA pressure results in increased PCWP and pulmonary venous hypertension. It has been shown that elevated PCWP during rest or exercise is inversely related to exercise performance136 and is correlated with mortality [135, 136]. Accordingly, reducing PCWP in HFpEF is a potential therapeutic target that may be achieved by diuretics and vasodilators, alternatively by a shunt from the left to the right atrium (RA). A number of devices and procedures have been developed to create a left to right shunt. The overall efficacy of the most recent REDUCE LAP-HF II Study was neutral [137]. Analysis of treatment on total HF events showed differential effects in pre-specified subgroups and identified pulmonary vascular function during exercise as the primary determinant for beneficial or harmful effects of the shunt. In a subsequent systematic post hoc statistical analysis, patients with normal exercise pulmonary vascular resistance (PVR ≤ 1.74 WU) without a pacemaker derived a significant clinical benefit from the shunt with a 55% reduction in the rate of HF events. The Kansas City Cardiomyopathy Questionnaire (KCCQ) overall summary score showed a 5.5-point higher increase from baseline in shunt patients, with 40% of shunt patients showing more than a 20-point increase [138]. Accordingly, the currently ongoing confirmatory Responder-HF study is a randomized, multicenter, double-blind, sham-controlled trial to evaluate clinical outcome in patients corresponding to the Responder cohort identified from the post hoc analysis of the REDUCE LAP-HF II study.

## Acute heart failure in patients with HFpEF

Acute HF is defined as a rapid or gradual worsening or onset of the HF syndrome urging patients to pursue medical attention [10]. Patients are referred to an emergency medical department or seek outpatient support. The prognosis of acute decompensated HFpEF and HFrEF is comparable [139]. Haemodynamic congestion, as shown with measurements of the diameter of the inferior vena cava and LA area, is comparable in acute decompensated HFpEF and HFrEF patients [140].

The recommended diagnostic work-up for new onset acute HF in HFpEF and HFrEF patients is similar [10] and outlined in Table 5. The basic therapeutic approach to acute HF in patients with HFpEF and HFrEF overlaps grossly and includes the administration of loop diuretics, vasodilators, inotropes, vasopressors, renal replacement therapy, and mechanical support as needed and where appropriate [10]. In the recently published ADVOR trial, acetazolamide was associated with a higher diuretic response and shortened length of hospital stay independent of LVEF [141]. In the ROPA-Dop trial, low-dose dopamine did not improve renal function in patients hospitalized for acute HFpEF, and continuous intravenous loop diuretic treatment was associated with worsening renal function compared to intermittent delivery [142]. Ultrafiltration was not effective in acute HF independent of LVEF. In fact, in those patients with an LVEF > 40%, ultrafiltration resulted in worsened renal function [143]. So far, no specific therapeutic approach has exclusively been established for acute decompensated HFpEF. The rehospitalization rate is higher in patients with previous HFpEF-related hospitalization and comparable to HFrEF patients with no previous HF-related hospitalization [139]. Interestingly, an SGLT2i was recently shown to reduce CV mortality and worsening HF in patients with and without previous HF hospitalization [144]. Beneficial effects of SGLT2i including enhanced diuresis and improved diuretic efficiency in subjects with acute decompensated HF seem to be independent of the underlying form of cardiomyopathy and include patients with and without preserved LVEF [127, 145, 146].

**Table 5:** Diagnostic work-up in patients presenting with acute HFpEF

Taking patient’s history and physical examination regarding signs and symptoms of HF
Electrocardiogram
Measurement of oxygen saturation
Echocardiography
Clinical chemistry including assessment of natriuretic peptides
Chest x-ray
Pleural and lung ultrasound

## Outlook

It is important to note that classification of HF according to LVEF is purely arbitrary and in its present form not based on physiological or clinical data. Historically, HFrEF was defined as LVEF < 35% that was later changed to < 40% to allow facilitated inclusion into clinical trials. Recently, two studies showed different behaviors of patients with HF symptoms [147, 148]. Patients with an LVEF > 60% showed a left shift of the pressure–volume relationship representing a form of contracture [148]. In contrast, patients with lower LVEF values < 60% shifted to the right with higher volumes at similar filling pressures representing the features of HFrEF.^150,151^ Therefore, another classification based on these pathophysiological studies has recently been proposed [149].

Given the complex pathophysiology and clinical presentation of HFpEF, the future of diagnosing and treating HFpEF lies in integrated approaches, combining data from multiple clinical and imaging modalities to provide a holistic view of the condition. The incorporation of artificial intelligence (AI) and machine learning in HFpEF imaging has the potential to revolutionize the field. AI-driven algorithms can help in identifying novel imaging biomarkers specific to HFpEF by analyzing large datasets from various imaging modalities [150, 151]. These advanced techniques can facilitate the automatic segmentation and quantification of cardiac structures, reducing the time and effort required for manual image analysis. AI can also be used to enhance the interpretation of echocardiographic and CMR images, leading to better diagnostic accuracy and more comprehensive risk assessment [152, 153]. These approaches might also inform about novel and individualized treatments of this heterogenous syndrome.

## Conclusions

The clinical diagnosis of HFpEF is a multifaceted process that relies on the integration of predisposing risk factors, clinical signs and symptoms, biomarkers, imaging findings, and exercise testing including CPET. The diagnosis HFpEF should not be rejected on the basis of normal natriuretic peptides levels in patients with unexplained dyspnoea, because this will lead to missed diagnoses and delayed treatments. The HFA-PEFF algorithm offers a practical and structured approach to aid clinicians in this diagnostic process. Accurate diagnosis is essential to guide management and treatment strategies, ultimately improving patient outcomes. Besides, it remains important to accurately identify and treat comorbidities contributing to the HFpEF syndrome. Advances in modern imaging techniques and the development of future tools hold great promise for improving the diagnosis and management of HFpEF. As our understanding of HFpEF evolves, so too will our ability to harness these cutting-edge technologies to benefit patients and optimize care.

## Abbreviations

ACC: American College of Cardiology
ACEi: Angiotensin converting enzyme inhibitor
AF: Atrial fibrillation
AHA: American Heart Association
AI: Artificial intelligence
ARB: Angiotensin-receptor blocker
ARIC: Atherosclerosis Risk In the Community
ARNI: Angiotensin-receptor neprilysin inhibitor
ATTRh: Hereditary transthyretin amyloidosis
ATTRwt: Wild-type transthyretin amyloidosis
BMI: Body mass index
BNP: B-type natriuretic peptide
CAD: Coronary artery disease
CKD: Chronic kidney disease
CMR: Cardiovascular magnetic resonance
COPD: Chronic obstructive pulmonary disease
CPET: Cardiopulmonary exercise test
CT: Computed tomography
CV: Cardiovascular
DM: Diabetes mellitus
DPD: Diphosphonate scintigraphy
ECV: Extracellular volume
eGFR: Estimated glomerular filtration rate
ESC: European Society of Cardiology
HF: Heart failure
HFmrEF: Heart failure with mildly reduced ejection fraction
HFpEF: Heart failure with preserved ejection fraction
HFSA: Heart Failure Society of America
HFrEF: Heart failure with reduced ejection fraction
HNCM: Hypertrophic non-obstructive cardiomyopathy
HOCM: Hypertrophic obstructive cardiomyopathy
ID: Iron deficiency
LA: Left atrial
LAVI: Left atrial volume index
LGE: Late gadolinium enhancement
LV: Left ventricle/ventricular
LVEDP: Left ventricular end-diastolic pressure
LVEF: Left ventricular ejection fraction
MR: Mitral regurgitation
MRA: Mineralocorticoid-receptors antagonist
NT-proBNP: N-terminal pro-B-type natriuretic peptide
PCWP: Pulmonary capillary wedge pressure
PET: Positron emission tomography
PVI: Pulmonary vein isolation
SGLT2: Sodium glucose transporter 2
STE: Speckle-tracking echocardiography
TDI: Tissue Doppler imaging
TR: Tricuspid regurgitation
TTE: Transthoracic echocardiography
TTVI: Transcatheter tricuspid valve intervention
